# Dual-process theories and consciousness: the case for ‘Type Zero’ cognition

**DOI:** 10.1093/nc/niw005

**Published:** 2016-05-09

**Authors:** Nicholas Shea, Chris D. Frith

**Affiliations:** ^1^ Department of Philosophy, King’s College London, Strand, London, WC2R 2LS, UK,; ^2^ Wellcome Trust Centre for NeuroImaging at UCL, University College London, 12 Queen Square London, WC1N 3BG, UK and; ^3^ Professorial Fellow, Institute of Philosophy, University of London, Senate House, Malet Street, London, WC1E 7HU, UK

**Keywords:** consciousness, unconscious processing, theories and models, function of consciousness, dual processing

## Abstract

A step towards a theory of consciousness would be to characterize the effect of consciousness on information processing. One set of results suggests that the effect of consciousness is to interfere with computations that are optimally performed non-consciously. Another set of results suggests that conscious, system 2 processing is the home of norm-compliant computation. This is contrasted with system 1 processing, thought to be typically unconscious, which operates with useful but error-prone heuristics. These results can be reconciled by separating out two different distinctions: between conscious and non-conscious representations, on the one hand, and between automatic and deliberate processes, on the other. This pair of distinctions is used to illuminate some existing experimental results and to resolve the puzzle about whether consciousness helps or hinders accurate information processing. This way of resolving the puzzle shows the importance of another category, which we label ‘type 0 cognition’, characterized by automatic computational processes operating on non-conscious representations.

## Introduction

What is consciousness good for? What special kinds of cognition does it enable? One prominent way of responding to this question is to challenge the presupposition that consciousness improves cognition – that consciousness is good for anything (Rosenthal 2008, see also Frith and Metzinger 2016). There are a range of experiments showing that precise and well-adapted behaviour can be produced without consciousness (Jacob and Jeannerod 2003; Goodale and Milner 2004). Furthermore, there are many cases where performance deteriorates when subjects become conscious of what they are doing (Beilock et al. 2002). From this perspective it can seem as if consciousness just gets in the way of the speed and efficiency – the optimality – of non-conscious processing.

By contrast, researchers relying on ‘dual processing’ to explain behaviour, often in the field of judgement and decision-making, draw a different conclusion about consciousness. They agree that automatic largely non-conscious type 1 (or system 1) processing is fast and can be heuristically useful, but they also show that, in a range of contexts, type 1 processing gets the wrong answer (Tversky and Kahneman 1974; Evans and Stanovich 2013). It is only with time and deliberate, conscious type 2 (or system 2) reasoning that subjects produce the correct or normative response (Kahneman 2003). When type 2 processing is compromised by cognitive load, subjects will fall into a range of type 1 errors like the conjunction fallacy, anchoring and base rate neglect.

So which is it? Does consciousness get in the way of accurate processing or facilitate it? If a subject becomes consciously aware of what they are doing, are they more likely to get it right or to get it wrong? In this article we resolve this seeming paradox by showing that different distinctions are in play in the two literatures. What at first look like two opposing positions about the function of consciousness, each supported by a substantial body of experimental results, can in fact be reconciled.

One distinction is about representations and the other is about processes. The *representations* over which cognitive processes operate can be conscious or non-conscious. Cognitive *processes* are another matter: they are computations from some representations to others. The process occurs ‘over’ the representations: cognitive processes occur through the unfolding of a sequence of representations. The type 1/type 2 distinction picks out two ways that cognitive processes can unfold (or, more likely, a continuum between two extremes). The process can be deliberately controlled and subject to cognitive load, or automatic and insensitive to cognitive load. By contrast the conscious/non-conscious distinction picks out two ways that representations can figure in cognition. They can be part of the conscious mental life of the subject, or not.

The apparent paradox we started with is dispelled when this distinction is made clear. Focusing on representations, there are indeed a number of paradigms in which conscious representations lead to different behavioural results than non-conscious representations, and in which a representation becoming conscious can lead to a departure from optimality. Focusing instead on processes, there are results showing that automatic, type 1 processing can produce incorrect performance in circumstances where, given more time or less cognitive load, subjects are able to meet a normative standard by engaging in type 2 processing. (Whether this is best theorized in terms of a distinction between two different systems, rather than two modes of operation of the same system, is a further question: Osman 2004; Kruglanski and Gigerenzer 2011; Oaksford and Chater 2014.) In short, the findings about conscious versus non-conscious representations are compatible with the findings about type 1 versus type 2 processes.

Once we have diffused the seeming paradox using these two distinctions it becomes clear we need a new category in our taxonomy. The type 1/type 2 distinction does not cover the whole territory. We will call the new category ‘type 0’ cognition. In type 2 cognition the input and output representations are conscious and the cognitive process that occurs over them is deliberate or controlled and susceptible to cognitive load. Most examples of type 1 processing in the literature also involve conscious representations. Subjects are usually responding to a consciously represented input (e.g. a mathematical question), and are usually conscious of what they say or do in response. What distinguishes type 1 from type 2 cognition is that the process that occurs over these representations is fast, automatic and relatively insensitive to cognitive load. To the extent that type 1 processing is characterized as non-conscious, that is usually because the subject is not conscious of the process by which they compute the output. The representations that make up the input and output of a type 1 process are, however, typically conscious. So here we characterize type 1 cognition as consisting of automatic processing of conscious representations.

Type 0 cognition combines both non-conscious representations and non-controlled processing. In type 0 cases the input representations are not conscious and the cognitive processing is automatic and insensitive to cognitive load. As we shall see, it is important to distinguish type 0 cognition from the bulk of the cases that have been given the label ‘type 1’ or ‘system 1’, because type 0 cognition has a different computational signature.

This article will make the case for picking out type 0 cognition as its own category. We show how various theoretical tangles, including our initial paradox, are resolved when we keep the two distinctions clear: the distinction between two types of representations – conscious versus non-conscious – on the one hand, and between two types of cognitive process – deliberate, typically slow, serial and affected by cognitive load versus automatic, typically fast, parallel and insensitive to cognitive load – on the other hand. Section 2 defines the difference between type 0 and type 1 cognition. Section 3 draws the contrast between type 1 and type 2 cognition. Section 4 puts these distinctions to work to understand two sets of existing findings.

## Conscious versus Non-conscious Representations: Type 1 versus Type 0

### What does consciousness facilitate?

We use ‘consciousness’ to mean both awareness and what-it’s-like-ness (i.e. both access and phenomenal consciousness: Block 2005). So a conscious representation forms part of a subject’s awareness in the sense that it is available for verbal report and use by other consuming systems: reasoning, selecting targets for action, storage in episodic or semantic memory, and perhaps other consuming systems at the personal level. Representing consciously also has a subjective character for the subject – it part of their phenomenal mental life.

There is plenty of evidence suggesting that consciousness, or an attentional phenomenon closely related to consciousness, is important for many forms of learning, memory and voluntary control of behaviour. However, a leading strategy in scientific research on consciousness is in search of something stronger: tasks that can *only* be performed using conscious representations. For example, it has been variously claimed that consciousness is required in order: to integrate or bind perceptual features (Dehaene and Naccache 2001; Baars 2002; Tononi 2004); to keep a representation online in the absence of stimulation (Greenwald et al. 1996; Dehaene and Naccache 2001) or to integrate motivational states with causal learning (Dickinson and Balleine 2009).

The history of this enterprise is not encouraging. Almost all proposed functions have been matched by plausible findings where the effect is shown to be produced in the absence of consciousness (Faivre et al. 2014; Soto et al. 2011 and Winkielman et al. 2005, respectively). Certainly, there is no clear case of a task for which consciousness is required. Disputes arise because of the intricacies of measuring consciousness and its absence – such that some researchers doubt that there is any non-chance performance without consciousness (Newell and Shanks 2014). So the case that there is actually any cognitive processing of non-conscious representations is far from conclusive (Phillips 2015).

In our view, it is entirely unsurprising that there is no clear case of a task for which consciousness is definitively required. It is misguided to think that the function of consciousness must consist in tasks that can only be performed with conscious representations. The motivation for that is the unobjectionable thought that consciousness facilitates certain kinds of cognitive process. But facilitation is not necessity. Consciousness may make a range of tasks easier. It would be much more demanding to expect any of these tasks to be strictly impossible to perform in any other way. When a particular task is performed in reliance on conscious representations, that works because computations take place over those representations so as to enable the subject to perform the task. There seems to be no reason in principle why the same computational steps could not be performed on non-conscious representations so as to produce a matching pattern of behaviour in the same task. In other words, even if consciousness functions to facilitate certain kinds of computations, and hence the performance of certain kinds of tasks, it would be odd – within a broadly computational/cognitive neuroscientific approach to the mind – if it were absolutely impossible to do the same task non-consciously.

The key idea here is facilitation. If consciousness has a distinctive function, that may indeed be because it is necessary for certain operations; or it may instead lie in facilitating a range of computational operations, each of which could in principle be performed on non-conscious representations. An analogy is the way that books can be arranged on a bookshelf. If they are ordered by subject and author that makes it much easier to perform a range of tasks: finding a particular book; finding all books on a particular subject; finding all books by a particular author; etc. However, each of those operations can be performed on a randomly ordered bookshelf. Similarly, we should be looking for a range of tasks that all tend to be facilitated when the representations involved are conscious. Finding that each of these tasks can be performed non-consciously does not, by itself, undermine the claim that consciousness facilitates the ability to perform all of them. If a representation’s being conscious has this kind of facilitatory effect, that is an important functional role of consciousness.

Although facilitating some computations, a representation’s being conscious may make other computations harder. So we may find that some computations that are performed optimally on non-conscious representations are performed sub-optimally when the representations become conscious (Levine et al. 1996). When an incoming representation is not conscious it may be relatively easy to update all the representations within the same informationally encapsulated module in a Bayes-optimal way. If the same new information is represented consciously it can potentially interact with everything else represented in any of the agent’s cognitive systems, making it difficult or impossible to compute a Bayesian update (Chater et al. 2006). So even if consciousness facilitates some computations, it may make others more difficult.

### Type 0 cognition

Type 0 cognition is characterized by automatic processes occurring over non-conscious representations. Models of reinforcement learning show how automatic processes can produce optimal behaviour in some domains. Subjects can learn how to behave so as to harvest near-maximum rewards when the contingencies between behaviour and rewards are probabilistic and changeable (O’Doherty et al. 2003). When presented with a situation, e.g. a pair of stimuli, subjects who have experienced a history of feedback on similar choices can decide which to choose very rapidly, and do so near optimally. These learning processes are automatic – they do not depend on the subject exercising deliberate control and are relatively insensitive to cognitive load (Otto et al. 2013). Making optimal choices in this way is computation-light but learning-heavy (Dayan 2014). This is the first of two examples of type 0 cognition that we discuss briefly in this section. We go on to point to some evidence that consciousness can impair the smooth operation of type 0 cognition.

We offered reinforcement learning as an example of an automatic process that relies on a history of feedback in order to make optimal choices in the present. If this kind of learning can occur over non-conscious representations, that would make it a case of type 0 cognition. And there is indeed good evidence that instrumental conditioning can take place on stimuli which are not consciously represented (Pessiglione et al. 2007, 2008). In short, given sufficient learning history in a domain, type 0 cognition can generate adaptive or near-optimal behaviour.

The second example is motor control. In many circumstances people adjust their ongoing movement on the basis of new information that arrives while they are executing an action (Goodale et al. 1986; Fourneret and Jeannerod 1998; Schindler et al. 2004). They do so in an optimal way using feedback control (Wolpert and Landy 2012). Subjects also compensate for their own visuomotor error in a near-optimal way (Zhang et al. 2015).

These rapid adjustments are made automatically, without deliberate control (Pisella et al. 2000). The adjustments are so rapid that it is highly unlikely that a representation of the new target location has become conscious by the time the adjustment is made. That is consistent with the finding that subjects can adjust to a target moved during a saccade without being aware that the target has moved (Fourneret and Jeannerod 1998). When subjects learn over repeated trials to adjust motor output to compensate for an artificial force field (Thoroughman and Shadmehr 2000), one kind of adjustment is also made automatically and without the subject being conscious of the adjustment (the slow implicit process in McDougle et al. 2015). There being an effect of non-conscious representations on perceptuomotor control in this way is consistent with the finding that perceptual learning about motion direction can occur without the direction of motion presented being consciously represented (T. Watanabe et al. 2001). In short, fine-grained online motor control rapidly performs complex computations over non-conscious representations, computations that meet a normative criterion.

Thus, model-free reinforcement learning can generate optimal decisions when making choices for rewards, and feedback control can compute optimal action trajectories. In both of these examples of type 0 cognition, non-conscious representation goes hand-in-hand with correct performance. Furthermore, when both systems make predictions about the same outcome, there is evidence that their outputs are integrated in a way that produces normatively appropriate behavioural output (O’Reilly et al. 2013).

Type 0 cognition is likely to play a large role in several other domains, for example in the rich inferences which occur automatically and without consciousness in the course of perception, language comprehension and language production.

Any input is potentially relevant to where to reach and whether to keep reaching. The fact that a colleague’s phone rings may mean, by a subtle chain of reasoning, that I should pick up the target object with the other hand. But the type 0 computations we have been discussing don’t make use of domain-general information. They are only performed on a very limited subset of incoming information (e.g. about the location and motion of the target) – information whose relevance to action execution subjects will have been able to learn about in the past. For example, the subjects in Pisella et al. (2000) performed rapid corrections in response to changes in target location but not in response to changes in target colour. The ‘automatic pilot’ mechanism of perceptuomotor control appears to be unable to make adjustments to take account of relevant colour information. We hypothesize that type 0 cognition is poor at integrating information from previously unconnected domains.

On the other hand, when representations become conscious behaviour may be less accurate than that produced by type 0 cognition. Subjects viewing a hollow face have a conscious experience that it is a normal convex face and make judgements about the location of small targets placed on the face accordingly. However, their rapid reaching movements are correctly targeted at the true location of the targets (i.e. further away than conscious experience represents them to be: Kroliczak et al. 2006). In line with the evidence above that rapid reaching movements are controlled by non-conscious representations of the target, it seems that type 0 cognition is able to drive correct performance in this context, while at the same time the judgements made on the basis of conscious representations are incorrect. There is also wider evidence that dorsal stream representations controlling action execution are not conscious (Jacob and Jeannerod 2003; Goodale and Milner 2004). The automatic post-error slowing observed in skilled typists may also be the result of type 0 cognition – it is independent at least of conscious visual representations of the letters produced (Logan and Crump 2010).

Similarly, consciousness or conscious attention can interfere with the smooth execution of actions controlled by type 0 cognition (Jueptner et al. 1997). For some high level sporting skills, the performance of expert practitioners is impaired when they attend more to what they are doing (Beilock et al. 2002). Motor skill learning can also be impaired by conscious reflection (Fletcher et al. 2005; McKay et al. 2015). In these cases conscious attention to aspects of the task appears to increase the processing demands and to lead to slower and less accurate performance. This is comparable to the way that performance on a task is impaired when the stimulus set or response set is increased (Hick 1952).

In short, type 0 cognition performs rapid and accurate computations on a limited range of information. It avoids the costs in speed and accuracy that would follow from bringing a wider range of information to bear on performing a task.

### Type 1 cognition

Type 1 cognition is characterized by automatic, load-insensitive processing of consciously represented inputs; outputs are typically also conscious. Experiments contrasting conscious with non-conscious processing suggest that processing in perceptual areas of the brain is not sufficient for a representation of a stimulus to become conscious in our sense. Conscious representation goes with activation of prefrontal cortex (particularly dorsolateral prefrontal cortex), usually together with inferior parietal cortex and anterior cingulate cortex (Dehaene et al. 2014). Our hypothesis is that, compared with type 0 cognition, consciousness facilitates the processing together of representations drawn from domains that have not previously been extensively associated.

It is hardly surprising that consciousness allows representations from previously unconnected domains to be integrated for computational processing, since by ‘conscious’ we mean (in part) access conscious. Access conscious representations are available to a range of personal-level consuming systems (verbal report, goal-directed reasoning, episodic memory storage, etc.). That is not equivalent to making information globally available, which may be a wider form of availability (e.g. to subpersonal systems), but our definition does require conscious representations to be reasonably widely available. A representation encapsulated in a domain-specific module, inaccessible to reasoning or verbal report, does not count as conscious as we are using the term. But nor would processing of a representation by deliberate reasoning on its own make a representation conscious, if that representation were not globally available.

Although some have claimed that consciousness is *necessary* for information integration (Singer 1998; Tononi 2008), the discussion in the section ‘What does consciousness facilitate?’ suggests that we should consider a more modest hypothesis: that consciousness facilitates information integration. And it does indeed appear that the necessity claim is too strong – multisensory integration can occur between subliminally presented stimuli (Faivre et al. 2014). However in the Faivre et al. (2014) paradigm, integration of non-conscious representations only occurred when participants had undergone previous conscious training on stimuli of the same kind. Prior consciousness thereby facilitated information integration. Indeed, it remains empirically open that, in the absence of the opportunity for extensive prior learning, people can only integrate non-conscious representations that have previously been associated consciously (Mudrik et al. 2014).

The role of consciousness in facilitating information integration can be seen in several paradigms in which local regularities are registered unconsciously but global regularities are only detected when stimuli are consciously represented. For example, subjects can extract a global pattern in incoming speech sounds when they are conscious (the fact that the sequence ‘aaaaB’ is repeated – Strauss et al. 2015). Sensitivity to violations of the global pattern is abolished in sleeping subjects, although sensitivity to violation of a local pattern remains (e.g. when a ‘B’ occurs in the sequence ‘aaaaB’: ‘passive sensory response adaptation’). Similarly, trace conditioning was abolished in one group of patients with disorders of consciousness; while delay conditioning, which is more local, was preserved (Bekinschtein et al. 2009).

A consequence of reduced informational encapsulation is that computations become more onerous. Bayesian inferences which can be performed in real time on a limited set of data can become computationally intractable as the range of information to be taken into account increases (Chater et al. 2006). Sometimes being able to behave even approximately correctly may require cognition to take account of information from separate domains (domains not experienced together much in the subject’s learning history). Consciousness may allow such processing, but the computational complexity of doing so makes it more likely that performance will be slow or incorrect.

So consciousness makes representations available to a wider range of processing, and processing that occurs over conscious representations takes a potentially wider range of representations as input. To reduce the resulting computational demands, the amount of information consciously represented about each stimulus or event may be reduced (e.g. Hillis et al. 2002).

For example, a situation that is non-consciously represented as a probability distribution over a range of possible values may collapse into a simpler representation when represented consciously (Stocker and Simoncelli 2008), e.g. of just a single value, or of a single value plus confidence. Stocker and Simoncelli (2008) found that once subjects had consciously decided that a stimulus was in one half of the screen, they effectively discarded probability information about the other half, cutting down the hypothesis space which was operative for subsequent decisions (see also Akaishi et al. 2014; Fischer and Whitney 2014). Along the same lines, Marcel (1980) found that ambiguous words presented unconsciously primed both meanings of the word, whereas consciously presented ambiguous words only primed the meaning that was consistent with prior context. In a similar way but at the neural level, Niv et al. (2015) found that a neural attentional control network did not represent all the perceptual dimensions presented to the subject, but only a variable task-relevant subset. (Interestingly, the reinforcement learning model that best explained subjects’ behaviour continued to include all nine available dimensions, suggesting that model-free reinforcement learning is a type 0 process that proceeds – in its limited way – without cutting down the representational space.) In all of these cases a stimulus’s being represented consciously, while making information about the stimulus available more widely, also goes along with a decrease in the amount of information being represented about the stimulus.

In short, type 1 cognition occurs over conscious representations, which has a benefit and also a cost. The benefit is access to a wider range of information and the ability to put together information without extensive prior experience. The cost is that optimal calculations may become computationally intractable, calling for a reduction in the amount of information carried and/or simplification of the processing being carried out.

## Automatic versus Deliberately Controlled Processes: Type 1 versus Type 2

### Type 1 cognition again

We saw in the last section that consciousness, which characterizes the representations involved in type 1 cognition, has computational costs. We suggested that this is sometimes dealt with by simplifying the computations to be performed. For example, rather than computing a full Bayesian belief update to take account of a new piece of information, heuristics may come into play. When heuristics are appropriate to the domain, these are the ‘simple heuristics that make us smart’ (Gigerenzer and Todd 1999) long thought to be characteristic of type 1 processing. But heuristics can of course go wrong. In such circumstances the greater time available to type 2 processing may be needed in order to compute the right answer.

Studies of the cognitive reflection test (Frederick 2005) show that people often use intuitive heuristics that produce the wrong answer to simple mathematical questions. (E.g. ‘A bat and a ball cost $1.10 in total. The bat costs $1 more than the ball. How much does the ball cost?’ – the intuitive answer, 10 cents, is incorrect.) In realistic everyday settings subjects’ priors appear to be sensitive and appropriate to varying contexts (e.g. cinema or cookery: Griffiths and Tenenbaum 2006), but type 1 cognition may get the wrong answer by applying priors that are inappropriate to the task domain (Fang et al. 2011). Conversely, in other circumstances the heuristics deployed in type 1 cognition get the correct answer where general purpose type 2 reasoning does not (Goldstein and Gigerenzer 1999; Gigerenzer and Sturm 2012).

In short, the heuristics and biases that characterize type 1 cognition are in part a response to the additional computational demands that come with conscious representation. In some but not all cases, given more time to solve the problem, deliberate reasoning is able to produce the correct answer where automatic reasoning does not (Kahneman 2003).

### Type 2 cognition

Type 2 cognition is characterized by deliberate, non-automatic processing of conscious representations. It is sensitive to cognitive load: type 2 processes interfere with one another. Type 2 cognition operates on conscious representations, typically in series, over a longer timescale than type 1 cognition. It can overcome some of the computational limitations of type 1 cognition, piecemeal, while retaining the advantage of being able to integrate information from previously unconnected domains. It is computation-heavy and learning-light: with its extended processing time, type 2 cognition can compute the correct answer or generate optimal actions without the benefit of extensive prior experience in a domain.

Deliberate reasoning tasks engage a large array of brain areas (Goel 2007), including the prefrontal and parietal areas mentioned above as associated with conscious representation. Examining deliberate reasoning of specific kinds brings out differences from the conscious–non-conscious contrast, with a left temporal lobe system involved in belief-based reasoning, and a bilateral parietal lobe system involved in logical or belief-neutral reasoning (Goel and Dolan 2003). The effect of increasing cognitive load also has a different neural signature. Cognitive load in general modulates the activity of superior parietal lobule and intraparietal sulcus bilaterally, and particularly of the right inferior frontal junction (Vergauwe et al. 2015). Thus, neural data are consistent with there being an important functional difference between our two distinctions (conscious-unconscious and automatic-deliberate).

One task that relies on type 2 cognition is being able to resist the automatic stem completion effect. Subjects are presented with a word stem and instructed to complete it in a way that differs from a prior masked word prime (e.g. if the prime is ‘table’ and the stem is ‘tab’ then ‘taboo’ would be a correct answer; ‘table’ would be incorrect). There is a tendency to complete the stem with the primed word. Subjects are able successfully to resist this tendency only if the prime was consciously represented (Debner and Jacoby 1994). This ability is compromised by cognitive load (Jacoby et al. 1993). Thus, successful performance of this task depends on type 2 cognition.

A second example is one of the two kinds of perceptual learning discovered by Schwiedrzik et al. (2011). They demonstrated two ways in which prior exposure to shapes can result in improved perceptual sensitivity. One kind of improvement does not require the stimuli to be consciously represented and occurs only in the retinotopic area in which they are presented. A second perceptual learning effect transfers to other retinotopic locations but only occurs when the stimuli are consciously represented. In similar perceptual learning tasks where there is a concurrent cognitive load (a letter identification task), improvements in sensitivity do not transfer to other retinotopic locations (Karni and Sagi 1991; see also K. Watanabe et al. 2006). Thus, the second type of perceptual learning in Schwiedrzik et al. (2011) is likely to rely on type 2 cognition whereas the first can occur with non-conscious stimuli in type 0 cognition.

Endogenous attention also seems to depend on type 2 cognition. It is susceptible to cognitive load (Jonides 1981). By contrast exogenous attention is relatively insensitive to cognitive load (Yantis and Jonides 1984) and can be driven by non-conscious stimuli (McCormick 1997). Those are then cases of type 0 cognition. Attention can affect the way the automatic processes of type 0 cognition are constrained to unfold (Naccache et al. 2002), as can other top-down effects (Yantis and Jonides 1990).

Importantly, type 2 cognition also allows deliberate productive language (speech), enabling an agent to select information to be communicated socially. For example, information broadcast deliberately as a result of type 2 metacognition may play an important role in joint action (Shea et al. 2014). It has recently been suggested that conscious representations of a thinker’s own agency, in particular the capacity to anticipate the regret that may be the result of performing an action, exemplify an important function of consciousness (Frith and Metzinger 2016). That too may be an important way that consciousness facilitates joint behaviour.

Deliberation is an important form of collective behaviour. When people communicate about a problem, they can often solve it together in a way that overcomes the limitations of individual type 1 cognition (Mercier and Sperber 2011; Maciejovsky et al. 2013). Since type 2 cognitive processes typically take place over a series of conscious representations, people can report about the steps involved (Ericsson 2006), and so can share expertise with others; whereas the intermediate steps of type 1 cognitive processes are usually opaque to the thinker (Nisbett and Wilson 1977; Berry and Broadbent 1984).

The distinction we draw between type 1 cognition and type 2 cognition has much in common with standard dual process accounts (system 1/system 2), except that the way we deal with consciousness is importantly different. System 1 processes have long been characterized as unconscious, and that remains a typical attribute even when it is not taken to be a defining feature (Evans and Stanovich 2013). As we characterize type 1 cognition, it takes conscious representations as input, and typically issues in conscious representations as output. It is our type 0 cognition that operates on non-conscious representations, using processing that has the features typically associated with system 1 (fast, automatic, load-insensitive, etc.).

Since our main disagreement with dual process theories concerns system 1, our type 2 cognition is very like standard views of system 2 processing. However, we do not assume that type 1 and type 2 are different systems or that they draw on qualitatively different kinds of cognitive processing. They may do, or they may instead reflect two poles of a continuum characterizing the way a single cognitive process can operate under more or less pressure.

Type 1 cognition and type 2 cognition deal in different ways with the demands imposed by the un-encapsulated nature of conscious representations. Type 1 processing uses cues and heuristics that enable rapid computations whereas type 2 cognition uses more time to deploy the more demanding and typically serial methods of general purpose reasoning. In some contexts a heuristic may outperform general purpose reasoning in accuracy as well as speed (Gigerenzer and Sturm 2012); in others the heuristic fails and type 2 cognition is needed to produce correct or norm-compliant behaviour.

## The Distinctions Applied to Existing Findings

### Model-based and model-free decision-making

Patterns of behaviour that have been learnt instrumentally in response to reward feedback have long been known to divide into goal-directed behaviour, which is immediately sensitive to the devaluation of an outcome, and habit-based behaviour, which is not (Dickinson and Balleine 1994, in non-human animals). Frith et al. (1992) found early evidence that the distinction is at work in humans: learning the behavioural rule needed to obtain a reward uses a separate system from learning the values of objects or stimuli. More recently, model-based reinforcement learning has been proposed to account for goal-directed behaviour and model-free reinforcement learning (discussed above) to account for habitual behaviour (Dayan 2014).

Model-based mechanisms learn about the connections between actions and outcomes and encode information about the causal structure of a task. Model-free mechanisms simply learn values for actions in each world state. In many settings this calls for considerable prior experience with the task, but makes it very straightforward to compute behavioural choices. Given a model of the situation, it is more complicated to calculate how to behave. For example, when the task is to make five binary choices in series to obtain a reward (e.g. in navigating around a maze) there are 32 possible sequences to consider. Subjects sometimes simplify the computation by reducing the problem space, cutting out some branches of the decision tree from consideration (‘pruning’), which can lead them to overlook the optimal sequence of choices (Huys et al. 2012).

We noted above that model-free reinforcement learning can take place on non-conscious representations. In those circumstances model-free learning is an instance of type 0 cognition. A rapid change in the stimulus-response mapping, on the other hand, seems to require that the stimuli are represented consciously (Pessiglione et al. 2011). These results are complicated by the fact that satiety has an effect on overall motivation, which will also affect behaviour driven by the model-free system. Ziauddeen et al. (2012) found that the overall level of motivation generated by a non-conscious stimulus (cumulative grip strength) is modulated by satiety in a food-specific way. They did not show that the stimulus-response mapping – which action is performed for each stimulus – activated by non-conscious stimuli is immediately modulated by reward devaluation (satiety).

Fear conditioning shows a similar signature, with two different systems at work. Subjects can be conditioned by electric shocks to acquire a fear reaction in response to stimuli represented non-consciously (Olsson and Phelps 2004). However, a verbal instruction that certain stimuli will produce a shock is only effective in producing a fear response when the stimulus is represented consciously (Olsson and Phelps 2004). (Cf. Pessiglione et al. (2011), although that effect may be due to the effect of subliminal stimuli on overall motivation.) So goal-directed learning and instructed rules seem to be beyond the reach of type 0 cognition.

Both the model-free and the model-based systems are at work in many reward-learning contexts (McNamee et al. 2015), with their relative reliability affecting the extent to which the model-based system gets control of behaviour (Donoso et al. 2014; Gershman et al. 2014; Lee et al. 2014). Cognitive load increases the extent to which a subject’s choice is driven by the model-free system (Otto et al. 2013). Individual differences in how much subjects exercise cognitive control in standard tasks predict the extent to which their choices are model-based in a reinforcement learning task (Otto et al. 2014). So it appears that model-based learning relies on type 2 cognition.

Interestingly, Economides et al. (2015) found that with extensive prior experience of task, model-based reasoning about what to choose becomes less susceptible to cognitive load (although it remains possible that the trained subjects switched to a different, non-model-based strategy, or that the effect of the training was to make the concurrent numerical Stroop task less load-inducing, cp. Flaudias and Llorca 2014). More generally, acting on social values seems to involve type 2 cognition: in the ultimatum game cognitive load makes subjects behave more in accordance with their personality type (Haruno et al. 2014).

Experiments on ego depletion and cognitive effort may also illuminate the extent to which type 2 cognition is able to influence behaviour. The relative influence of the model-based and model-free learning systems on behaviour, depending on their respective reliability, reflects a cost-benefit trade off. Similarly, the opportunity cost of devoting type 2 cognition to a problem should affect our willingness to rely on it. The feeling of cognitive effort accompanying some tasks may reflect that opportunity cost (Westbrook and Braver 2015). Thus, subjects given the suggestion that a strenuous mental task will be energizing showed less of an interference effect in a subsequent Stroop task (a standard measure of ego depletion) (Job et al. 2010). The effects of beliefs about free will may work in a similar way. Subjects given messages suggesting there is no free will show reduced preconscious motor preparation (Rigoni et al. 2011) and reduced intentional inhibition of a prepotent response (Rigoni et al. 2012). This may work by inducing subjects to reduce their estimate of the efficacy or reliability of type 2 cognitive processes, thus reducing their effect on behaviour.

In this section, we have contrasted model-based with model-free learning, briefly mentioning three other categories related to the model-based system (deploying a rule, exercising mental effort, exercising cognitive control). We argued that model-based decision-making depends on type 2 cognition whereas model-free decision-making can be performed by type 0 cognition.

### Confidence judgements

Confidence judgements as studied in cognitive psychology are found to be an only moderately reliable predictor of the subject’s accuracy in a task (Koriat 2012). By contrast, cognitive neuroscience often finds neural signals of confidence that are highly predictive of performance (Kepecs et al. 2008; Kiani and Shadlen 2009). This discrepancy is explicable if different cognitive systems are at work in the two cases. Internal measures of confidence can be based directly on features of the evidence used to make a decision or on the variance in a perceptual signal (Yeung and Summerfield 2012). Such signals can feed directly into the automatic computations of type 0 or type 1 cognition (e.g. Ernst and Banks 2002). Since noise is the main source of inaccuracy in these paradigms, it is unsurprising that confidence signals based on noise are good predictors of accurate performance.

By contrast the verbal confidence judgements elicited in cognitive psychological work on metacognition tend to be inferred from consciously represented cues like similarity and fluency (Thompson et al. 2011; Koriat 2012). These cues are relied on to form a judgement as to how likely it is that the type 1 heuristic used to solve a problem will get it right. Since the cues are only indirect and approximate indicators of likely accuracy, confidence judgements based on them are less reliable. Vlassova et al. (2014) found that subjects produce explicit metacognitive reports, as assessed by meta-d’, only on the basis of consciously represented aspects of a stimulus. Unconsciously represented information had an effect in facilitating task performance but without affecting meta-d’. This suggests that explicit reports of confidence depend on type 2 cognition.

## Conclusion

We started by asking what consciousness is good for – what kinds of cognition does it enable? To return to that question: does consciousness help us get things right or does it make us get them wrong? The article argues that we should divide that question into two separate questions, one about processes (deliberate vs. automatic) and the other about representations (conscious vs. non-conscious). In familiar domains, automatic processing of non-conscious representations can generate impressively near-optimal performance (type 0 cognition). A representation’s being conscious can facilitate some computations that are otherwise difficult, for example by allowing information to be integrated from domains not previously encountered together. However, that also makes the computations less tractable, which can require computational shortcuts or heuristics, which in turn can lead to incorrect performance when relied on outside the context they were designed for (type 1 cognition). Those limitations can be overcome in some cases by our limited-capacity ability to engage in step-by-step deliberate reasoning (type 2 cognition).

Two distinctions that have often been run together using the conscious–unconscious contrast can usefully be separated, giving us a more accurate way of dividing up existing experimental results. However, a 2 × 2 distinction generates four possibilities, and we have only discussed three (type 0, 1 and 2 cognition: see Table 1). What of the fourth box? This would be the home of deliberate processes acting on non-conscious representations. It seems to us that there may well be no type of cognition that fits in this box. If so, that is an important discovery about the nature of consciousness.

**Table 1.** Types of cognition located by reference to the two distinctions discussed in the text

		Processing	
		Automatic	Deliberate
Representations	Non-conscious	Type 0 cognition	?
	Conscious	Type 1 cognition	Type 2 cognition

From one perspective, it is unsurprising if deliberate reasoning cannot act on non-conscious representations. We defined consciousness as requiring access consciousness, i.e. the availability of a representation to verbal report and other ‘consuming systems’ of the whole person, like a person’s capacity for deliberate reasoning. So consciousness in our sense implies availability to deliberate reasoning. That definitional point does not alone secure the converse: that deliberate reasoning can only be performed on conscious representations. So that would be a substantive discovery. Furthermore, it is far from obvious that cognitive processes performed on subliminal stimuli should always be invulnerable to cognitive load. Nor that processing of subliminal stimuli should exert no cognitive load. If those predictions turn out to be confirmed empirically, then we will have discovered that the connection between deliberate reasoning and consciousness is remarkably tight. (Which may in turn explain why theorists have tended to run them together.)

We should distinguish two different reasons why the fourth box may be empty, corresponding to two strengths of claim about the function of consciousness: that it facilitates or is necessary for certain computations (see section: ‘What does consciousness facilitate?’). The first option is that there are few cases of deliberate reasoning on non-conscious representations because global broadcast has a facilitatory effect. Without a dedicated input domain of its own, deliberate reasoning tends only to take place on globally broadcast representations. Facilitation is compatible with it being possible for deliberate reasoning to take place over non-conscious representations (cp. Soto et al. 2011). Being processed by deliberate reasoning is not on its own sufficient to render a representation conscious, since consciousness requires that the representation be globally available.

The second option is that consciousness is indeed necessary for deliberate reasoning. One reason why there could be a constitutive connection between conscious representation and deliberate reasoning is if deliberate reasoning is constituted by a series of intermediate steps where each representation that concludes one step and starts the next step is represented consciously. Each step, taken alone, could be automatic, in which case it would be of type 1. Type 2 cognition would then be constituted by a series of steps of type 1. The effect of load could be due to the limited capacity of consciousness, rather than because of an impact on the performance of any of the automatic steps. On this picture it is because only a limited amount of information can be represented consciously at any one time that type 2 cognitive processes are affected by cognitive load.

It is an open empirical question whether cases in the fourth box are merely rare or missing entirely. In any event, the paucity of reports of load-sensitive, deliberate reasoning taking place over non-conscious representations suggests that there is a tight connection of some kind between deliberate reasoning and consciousness.

While there may be a tight connection when we are dealing with deliberate processing, this article has shown that there are strong reasons to think that automatic processing divides into two importantly different kinds: that performed on conscious representations (type 1 cognition) and that performed on non-conscious representations (type 0 cognition). Recognizing this difference not only allows for a clearer understanding of cognitive processes; it also allows us to see more clearly what the effect of consciousness on cognitive processes is – which is one small step towards constructing a theory of consciousness.
